# Primary mediastinal large B cell lymphoma in a woman who is human immunodeficiency virus positive presenting with superior vena cava syndrome: a case report

**DOI:** 10.1186/s13256-017-1200-z

**Published:** 2017-02-11

**Authors:** Pedro Pallangyo, Paulina Nicholaus, Frederick Lyimo, Elikaanany Urio, Peter Kisenge, Mohamed Janabi

**Affiliations:** 1 Department of Cardiovascular Medicine, Jakaya Kikwete Cardiac Institute, P.O. Box 65141, Dar es Salaam, Tanzania; 2 Department of Radiology, Muhimbili National Hospital, P.O. Box 65000, Dar es Salaam, Tanzania

**Keywords:** Mediastinal large B cell lymphoma, Non-Hodgkin lymphoma, HIV infection, AIDS, Superior vena cava syndrome, Case report

## Abstract

**Background:**

The risk of non-Hodgkin lymphoma is increased 200-fold in individuals seropositive for human immunodeficiency virus compared to those free from human immunodeficiency virus. Human immunodeficiency virus-associated non-Hodgkin lymphoma is known for its atypical presentation, aggressive ability, widespread involvement, poor response to chemotherapy, and high relapse potential which makes both the diagnosis and management a difficult undertaking especially in resource-poor settings.

**Case presentation:**

We report a case of primary mediastinal large B cell lymphoma in a 46-year-old woman of African descent who is human immunodeficiency virus positive who presented with symptoms of superior vena cava syndrome. Her past medical history was remarkable for a 23-year history of systemic hypertension and a 10-year history of human immunodeficiency virus infection. A physical examination revealed an underweight woman with right-sided facial, neck, upper limb, and trunk swelling together with distended veins on her chest and abdomen draining downwards. A respiratory examination revealed a reduced chest expansion, stony dull percussion note, and absent breath sounds on her entire right side with a left-sided tracheal deviation. She had a CD4 count of 146 cells/μL. A chest X-ray revealed a homogenous opacification on her right side with a left-sided tracheal deviation while a computed tomography scan of her chest revealed a solid mass on her right side. An echocardiogram showed a huge well-circumscribed mass (4.6×3.3 cm) with spontaneous echocardiographic contrast compressing her heart inferiorly. She had severe pulmonary hypertension (right ventricular systolic pressure 58 mmHg) but preserved left ventricular systolic function, no thrombus was seen, and her pericardium was normal. A computed tomography angiography of her aorta ruled out an aortic aneurysm. Finally, she underwent mediastinoscopy and a direct biopsy of the mass was taken for histopathology. Hematoxylin and eosin staining demonstrated a dense lymphoid infiltrate of large malignant cells with pleomorphic nuclei in clusters, compartmentalized by fine bands of fibrosis, and frequent mitoses were present. A diagnosis of mediastinal large B cell lymphoma was reached.

**Conclusions:**

The presence of a mediastinal widening coupled with a history of unintentional yet significant weight loss in an individual who is human immunodeficiency virus seropositive should raise an index of suspicion for lymphomas and warrant aggressive investigations and timely management.

## Background

The lung and mediastinum are often targets of human immunodeficiency virus (HIV) infection and severe thoracic complications are not uncommon. Differentiating the respiratory clinical manifestations of acquired immunodeficiency syndrome (AIDS) as infectious or neoplastic may pose a diagnostic challenge especially in resource-limited settings. Non-Hodgkin lymphoma (NHL) is among the AIDS-defining illnesses and in approximately 5 % of cases it is the initial manifestation of AIDS [1]. The risk of NHL is increased 200-fold in individuals infected with HIV compared to those free from it and up to 14 % of patients with AIDS with comorbid NHL present with a pulmonary involvement [2, 3].

Diffuse large B cell lymphomas (DLBCLs) are the commonest lymphomas in an HIV setting. Mediastinal large B cell lymphoma (MLBCL) is a subtype of DLBCL arising in the mediastinum. MLBCL is fast growing and aggressive usually presenting with symptoms of superior vena cava syndrome and a localized mediastinal mass with characteristic morphologic and genotypic features [4]. The introduction of highly active antiretroviral therapy (HAART) has resulted in longevity of life and a decreased incidence of lymphomas; however, the overall survival of patients who are HIV seropositive with NHL remains inferior to the survival of their general population counterparts [5]. We report a case of primary MLBCL in a 46-year-old woman of African descent who is HIV positive who presented with symptoms of superior vena cava syndrome.

## Case presentation

**Fig. 1:**
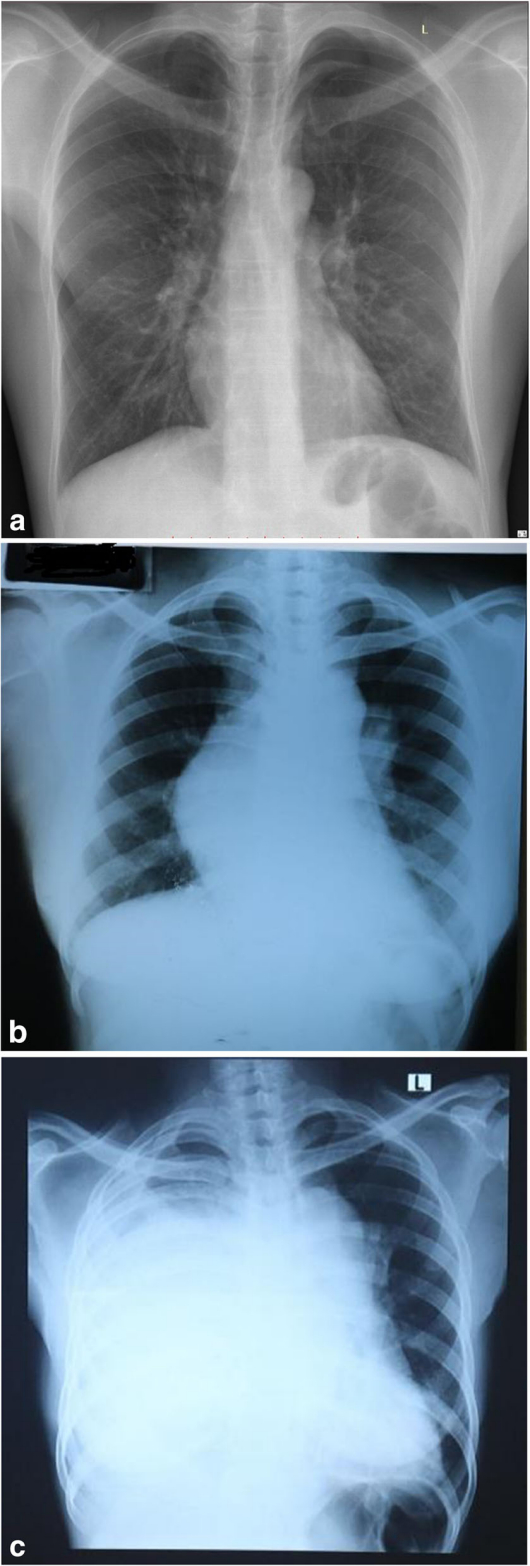
**a** Chest X-ray (posteroanterior view) taken 35 weeks prior, showing normal findings. **b** Chest X-ray (posteroanterior view) taken 21 weeks prior, showing right-sided mediastinal widening. **c** Chest X-ray (posteroanterior view) taken during the index admission, displaying right-sided homogenous opacification and left-sided tracheal deviation

A 46-year-old woman of African descent with a 23-year history of systemic hypertension and a 10-year history of HIV infection on HAART, with an unconfirmed prior AIDS-defining illness was referred to our institution by an upcountry regional hospital. She presented with chief complaints of right-sided facial, neck, upper limb, and trunk swelling together with shortness of breath for 35 days. Her right-sided swelling was of gradual onset and progressive with no identified aggravating or relieving factors. The swelling was associated with difficulty in swallowing, however, she denied any history of associated pain, numbness, color or temperature changes, or impaired vision. She denied any history suggestive of renal dysfunction, oral aphthous ulcers, or candidiasis. Shortness of breath was of gradual onset and worsened with increased right-sided swelling to the extent that regular activities, including walking and speaking, were impaired. It was associated with a dry cough; however, she denied any history of chest pain, night sweats, fever, or symptoms of heart failure. She reported an unintentional weight loss of over 15 kilograms within the past 1 year. She has neither smoked cigarettes nor worked or lived in or near an asbestos company. She reported that she had been diagnosed as having pulmonary tuberculosis (TB) based on radiographic features a year ago and completed a 6-month course of first-line anti-TB drugs; however, she did not present with documents indicating so. Her last hospitalization was 35 weeks prior when she had an episode of expectorating blood-stained sputum; however, her chest X-ray at that time displayed normal findings (Fig. 1a). Another chest X-ray with unidentified indication was done 21 weeks ago and revealed a right-sided mediastinal widening (Fig. 1b). Her CD4 count 29 weeks prior was 743 cells/μL and her last CD4 count 7 weeks prior to the index admission was 284 cells/μL. She was on a zidovudine, nevirapine, and lamivudine regime for approximately 9 years and was then switched to a tenofovir, lamivudine, and efavirenz regime in the past 11 months. The reasons for the regime switch were not documented, however, she reported good adherence throughout. Her husband and daughter of 28 months died of AIDS 10 years ago.

A physical examination revealed an underweight woman with a body mass index (BMI) of 16.8 kg/m^2^, with right-sided facial, neck, upper limb, and trunk swelling together with distended veins on her chest and abdomen draining downwards. Her blood pressure was 138/90 mmHg, respiratory rate 22 breaths/minute, oxygen saturation 97 % on room air, and she had a temperature of 37.1 °C. A respiratory examination revealed reduced chest expansion, stony dull percussion note, and absent breath sounds on her entire right side with a left-sided tracheal deviation. An apex beat was felt at the ninth intercostal space along the mid clavicular line and auscultation revealed a murmur of tricuspid regurgitation. A local breast examination revealed an edematous right breast that was otherwise normal. A musculoskeletal assessment revealed reduced muscle bulkiness in all four limbs but normal tone, power, and reflexes. The lymph nodes in her cervical, axillary, and femoral regions were not palpable.

**Fig. 2:**
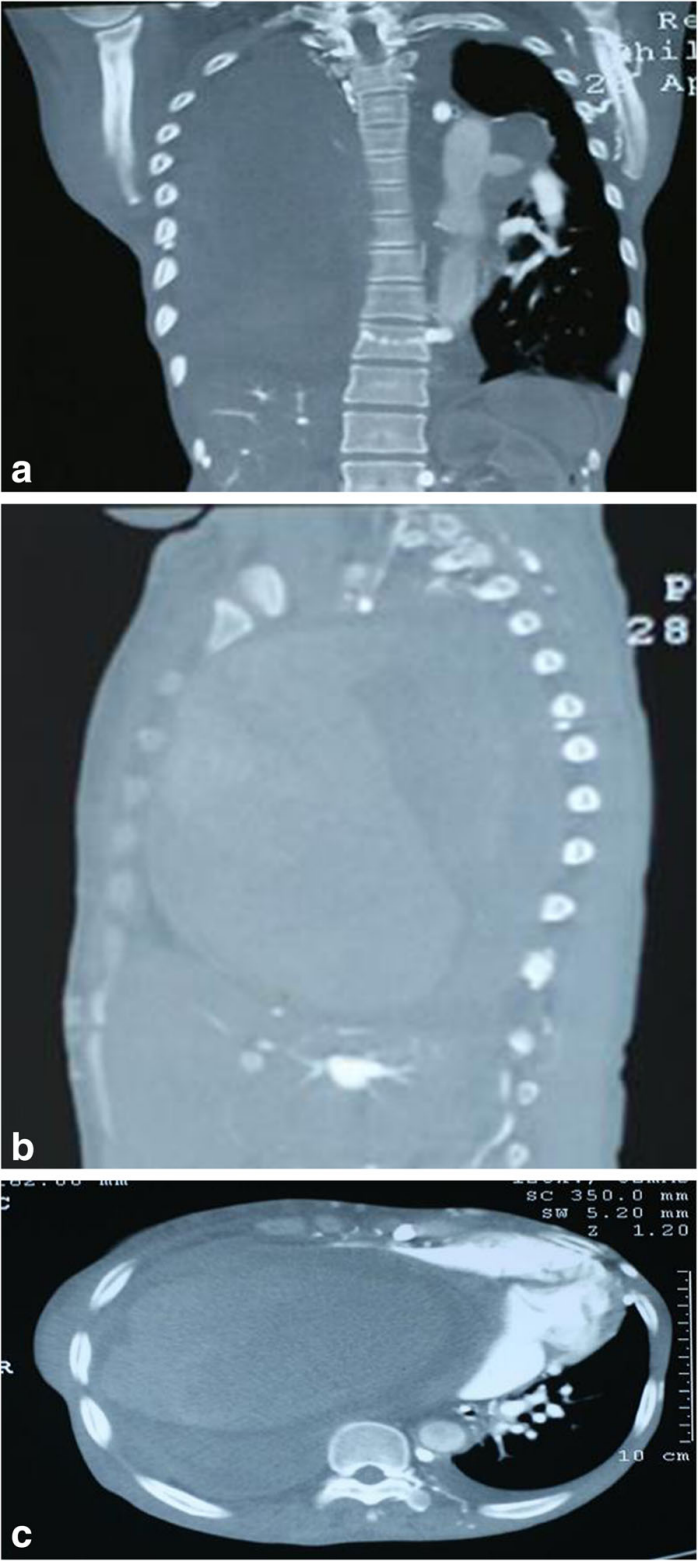
**a** Computed tomography chest (coronal view) displaying a huge right-sided mass. **b** Computed tomography chest (right parasagittal view) displaying a huge mass. **c** Computed tomography chest (axial view) displaying a huge right-sided chest mass causing mediastinal shift to the contralateral side

**Fig. 3:**
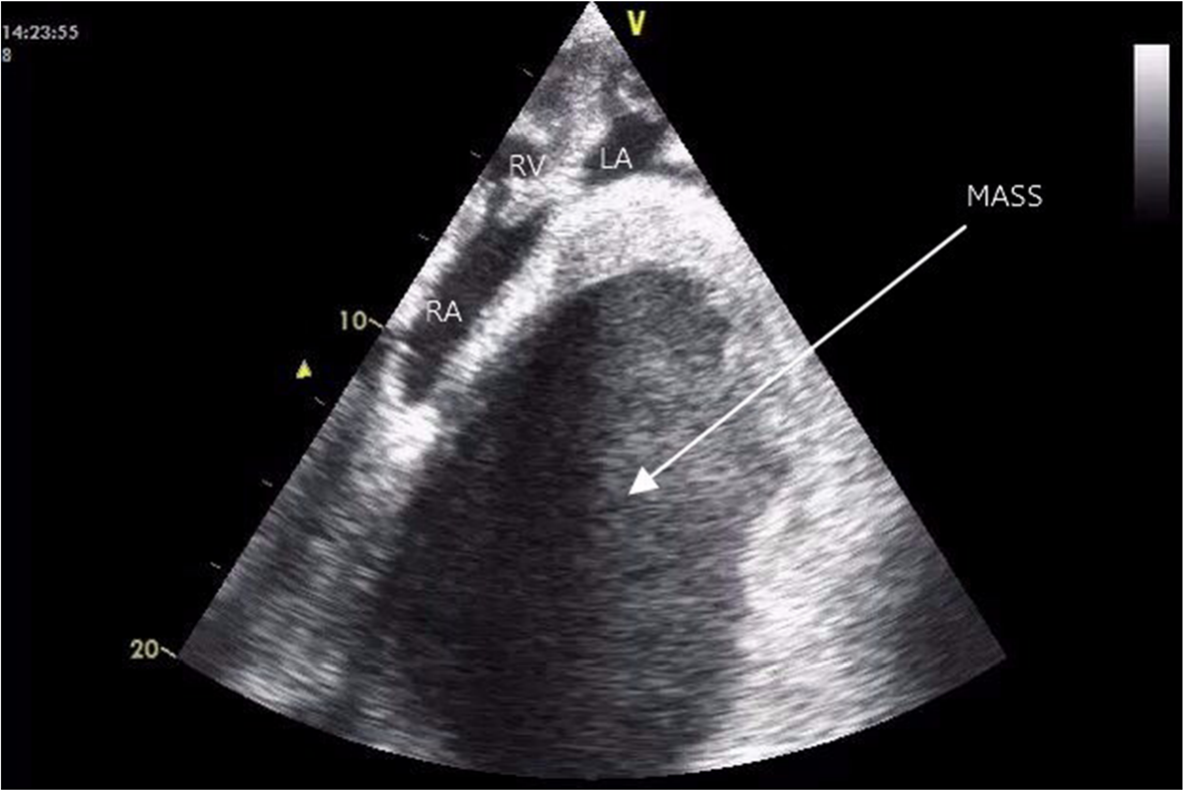
Echocardiogram (apical four-chamber view) displaying a huge mass (4.6×3.3 cm) with spontaneous ECHO contrast (arrow) compressing the heart inferiorly

She underwent a series of investigations which revealed anemia of iron deficiency: hemoglobin (Hb) 8.17 g/dL, mean corpuscular volume (MCV) 79.8 fL, mean corpuscular hemoglobin (MCH) 24.4 pg/cell, and random distribution of red cell width (RDW) 17.1 fL; however, she had normal renal and liver functions, lipids, electrolytes, and bleeding profiles. The Venereal Disease Research Laboratory (VDRL) test for syphilis, serology for hepatitis B and C, and autoimmune screening using antinuclear antibody (ANA) and antineutrophil cytoplasmic antibody (ANCA) were all negative. *Mycobacterium tuberculosis* was not detected by a GeneXpert test and her present CD4 count is 146 cells/μL. A chest X-ray revealed a homogenous opacification on her right side with a left-sided tracheal deviation (Fig. 1c), and a computed tomography (CT) scan of her chest revealed a solid mass on her right side (Fig. 2). An abdominal ultrasound revealed a right-sided pleural effusion and hepatomegaly (liver span 16.2 cm). An echocardiogram showed a huge well-circumscribed mass (4.6×3.3 cm) with spontaneous echocardiographic contrast compressing her heart inferiorly. There was severe pulmonary hypertension, that is, her right ventricular systolic pressure (RVSP) was 58 mmHg, but she had preserved left ventricular systolic function with an ejection fraction (EF) of 55 %; no thrombus was seen, and her pericardium was normal (Fig. 3). A CT angiography of her aorta ruled out an aortic aneurysm. Finally, she underwent mediastinoscopy and a direct biopsy of the mass was taken for histopathology. Hematoxylin and eosin staining demonstrated a dense lymphoid infiltrate of large malignant cells with pleomorphic nuclei in clusters, compartmentalized by fine bands of fibrosis, and frequent mitoses were present. Such biopsy findings are in keeping with a diagnosis of MLBCL. A bone marrow biopsy showed no evidence of lymphoma.

Although the diagnosis was finally reached, she died of respiratory failure a few days before the biopsy results were available.

## Discussion

Lymphomas are the second commonest malignancies after Kaposi’s sarcoma in the HIV-infected subpopulation. It is estimated that one out of five patients who are HIV seropositive will develop NHL in their lifetime [6]. HIV-associated NHL and in particular DLBLC are known for their atypical presentation, aggressive ability, widespread involvement, poor response to chemotherapy, and high relapse rates which complicates both the diagnosis and management [7]. The mediastinum is a common extranodal but a rare primary site for AIDS-related NHL and the occurrence of such malignancy is almost always an indicator of advanced HIV infection. Distinctively, HIV-related MLBCL displays a lower frequency of mediastinal adenopathy compared to its HIV-free counterparts. Although fatal if not treated, MLBCL is often curable with intensive chemotherapy combinations including Cytoxan (cyclophosphamide), Adriamycin (doxorubicin), vincristine, and prednisone (CHOP). The extent of disease, and extranodal and bone marrow involvement are key prognostic indicators in AIDS-related NHL and the reported median survival is 8 months [8].

In the case presented, she was known to be seropositive for HIV for approximately a decade with a self-reported good adherence to HAART. It is to our understanding that she started developing respiratory symptoms at least 5 months before she was referred to us. Moreover, she had experienced unintentional but significant weight loss for a couple of months’ duration. Disappointingly, despite evidence of a widened mediastinum not seen in the previous chest film taken 14 weeks prior, the attending clinicians did not investigate the cause of her mediastinal widening. It took a further 21 weeks before a lymphoma diagnosis was suspected and 3 additional weeks for the biopsy results to become available, at such time she had already died of respiratory failure. It is evident that several conditions manifest atypically in individuals who are HIV positive but this should sensitize clinicians to be even more aggressive in searching for the definitive diagnosis. Arguably, if the diagnosis of lymphoma was made during the initial presentation 24 weeks earlier this patient could have potentially benefited from chemotherapy with an ultimate increased survival.

## Conclusions

In conclusion, the presence of a mediastinal widening coupled with a history of unintentional yet significant weight loss in an individual who is HIV seropositive should raise an index of suspicion for lymphomas and warrant aggressive investigations and timely management.

## Abbreviations

AIDS: Acquired immunodeficiency syndrome
ANA: Antinuclear antibody
ANCA: Antineutrophil cytoplasmic antibody
BMI: Body mass index
CHOP: Cytoxan (cyclophosphamide), Adriamycin (doxorubicin), vincristine, and prednisone
CT: Computed tomography
DLBCL: Diffuse large B cell lymphoma
EF: Ejection fraction
HAART: Highly active antiretroviral therapy
Hb: Hemoglobin
MCH: Mean corpuscular hemoglobin
MCV: Mean corpuscular volume
MLBCL: Mediastinal large B cell lymphoma
NHL: Non-Hodgkin lymphoma
RDW: Random distribution of red cell width
RVSP: Right ventricular systolic pressure
TB: Tuberculosis
VDRL: Venereal Disease Research Laboratory
